# Comparative Transcriptome Analysis Unravels the Response Mechanisms of *Fusarium oxysporum* f.sp. *cubense* to a Biocontrol Agent, *Pseudomonas aeruginosa* Gxun-2

**DOI:** 10.3390/ijms232315432

**Published:** 2022-12-06

**Authors:** Shuyan Li, Junpeng Ma, Shiyong Li, Fuhui Chen, Chaodong Song, Hongyan Zhang, Mingguo Jiang, Naikun Shen

**Affiliations:** 1 Guangxi Key Laboratory of Polysaccharide Materials and Modification, School of Marine Sciences and Biotechnology, Guangxi Minzu University, Nanning 530000, China; 2 Guangxi Key Laboratory of Microbial Plant Resources and Utilization, School of Marine Sciences and Biotechnology, Guangxi Minzu University, Nanning 530008, China; 3 Guangxi Key Laboratory of Forest Products Chemistry and Engineering, Guangxi Minzu University, Nanning 530000, China

**Keywords:** banana *Fusarium* wilt, *Pseudomonas aeruginosa*, biocontrol, response mechanism, transcriptome analysis

## Abstract

Banana *Fusarium* wilt, which is caused by *Fusarium oxysporum* f.sp. *cubense* Tropical Race 4 (FOC TR4), is one of the most serious fungal diseases in the banana-producing regions in east Asia. *Pseudomonas aeruginosa* Gxun-2 could significantly inhibit the growth of FOC TR4. Strain Gxun-2 strongly inhibited the mycelial growth of FOC TR4 on dual culture plates and caused hyphal wrinkles, ruptures, and deformities on in vitro cultures. Banana seedlings under pot experiment treatment with Gxun-2 in a greenhouse resulted in an 84.21% reduction in the disease. Comparative transcriptome analysis was applied to reveal the response and resistance of FOC TR4 to Gxun-2 stress. The RNA-seq analysis of FOC TR4 during dual-culture with *P. aeruginosa* Gxun-2 revealed 3075 differentially expressed genes (DEGs) compared with the control. Among the genes, 1158 genes were up-regulated, and 1917 genes were down-regulated. Further analysis of gene function and the pathway of DEGs revealed that genes related to the cell membrane, cell wall formation, peroxidase, ABC transporter, and autophagy were up-regulated, while down-regulated DEGs were enriched in the sphingolipid metabolism and chitinase. These results indicated that FOC TR4 upregulates a large number of genes in order to maintain cell functions. The results of qRT-PCR conducted on a subset of 13 genes were consistent with the results of RNA-seq data. Thus, this study serves as a valuable resource regarding the mechanisms of fungal pathogen resistance to biocontrol agents.

## 1. Introduction

Banana *Fusarium* wilt is the most serious soilborne fungus disease caused by *Fusarium oxysporum* f.sp. *cubense* (FOC TR4), which has a wide range of occurrence and is difficult to control [1]. It invades the xylem tissues of roots and spreads through the vascular system of pseudostems, causing plant death. In severe cases, there is significant banana yield reduction or even extinction [2]. In recent years, banana wilt disease has occurred on a large scale in banana planting areas, and it has caused a devastating blow to the banana planting industry in China [3].

Despite the successful use of chemical fungicides to control banana wilt and increase yield, this method also causes environmental pollution and health problems. Biological control can effectively control banana wilt and inhibit pathogen growth through the use of microorganisms or their secondary metabolites. Many *Bacillus* and *Pseudomonas* strains have been widely investigated and used because of their direct or indirect beneficial effects of inhibiting FOC TR4. Kavita et al., found that strain CSR-D4 *Bacillus licheniformis* exhibited a high inhibition percentage (77.59%) against the FOC TR4 pathogen in a dual plate assay [4]. Siderophore production strain *P. aeruginosa* FP6 inhibited *R. solani* in the absence of FeCl_3_ (72.25%), which was reduced to 12% in the presence of FeCl_3_, suggesting siderophore-mediated antagonism [5]. Christopher et al., testified that wild-type *P. aeruginosa* can reduce *Phytophthora infestans* populations in the rhizosphere and roots of potato plants and reduce in planta disease symptoms caused by phenazine-1-carboxylic acid (PCA) production [6]. Current antagonistic microorganisms mainly include *Bacillus* sp. and *Pseudomonas* sp.; *Bacillus* sp. inhibit the fungal phytopathogens primarily through the secretion of lipopeptide, protease, and chitinase [7], while *P. aeruginosa* participates by producing siderophore, phenazine, pyocyanin, and extracellular polysaccharides.

The current research study mainly focuses on the mechanism of biocontrol bacteria, including the synthesis of antifungal activity substances, competition for survival factors with the pathogen, and the promotion of host plant defenses [8,9]. However, the complex response mechanism of the pathogen, whether it can resist or alleviate the stress of biocontrol agents, and the specific mechanisms thereof remain unknown. Transcriptome analysis offers a new strategy to clarify such mechanisms. Liu et al., revealed the response mechanism of *Valsa mali* to the strain Hhs.015 through comparative transcriptome analysis. In response to the inhibitory effects of Hhs.015, *V. mali* upregulated the synthesis and metabolism of amino acids, the synthesis and repair of the cell wall and cell membrane, as well as detoxification- and antioxidant-related genes [10]. Sun et al., found that *Bacillus velezensis* L-1 impaired the cellular structure of *Cytospora mali* QH2 and reduced the expression of virulence-related genes in *C. mali* QH2. At the same time, *C. mali* QH2 enhanced genes that are related to detoxification and antioxidation [11]. *P. aeruginosa* Gxun-2 has been isolated from healthy banana rhizosphere soil, and it effectively inhibited FOC TR4. In addition, it had significant antifungal activity against *Colletotrichum musae*, *Bipolaris sorokiniana*, *F. pseudograminearum*, *Alternaria alternata*, *Rhizoctonia solani*, and *Plectosphaerella cucumerina* due to the secretion of PCA and siderophore or other antifungal activity metabolites. However, the response mechanism of *F. oxysporum* to *P. aeruginosa* stress has not been reported.

In the present study, *P. aeruginosa* Gxun-2 and FOC TR4 were used as the biocontrol microorganisms and the plant pathogen interaction system. Transcriptome sequencing technology was used to compare the gene expression characteristics of the wild-type FOC TR4 and the FOC TR4 treated with *P. aeruginosa* Gxun-2 in order to reveal the response mechanism of FOC TR4 to Gxun-2 stress at the transcriptome level.

## 2. Results

### 2.1. Effects of P. aeruginosa Gxun-2 on the Growth and Morphology of FOC TR4

**Figure 1:**
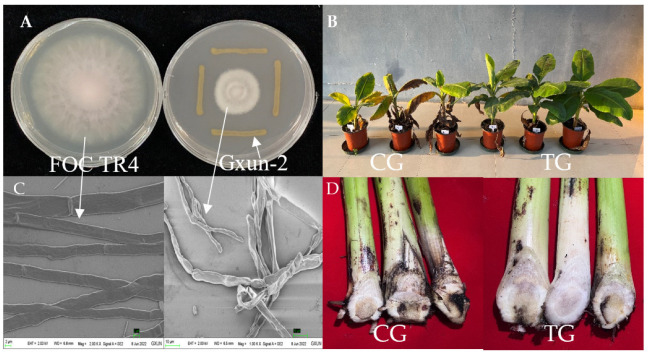
Antagonistic effects of Gxun-2 strains against *Fusarium oxysporum* f.sp. *cubense* (FOC TR4). (**A**) Gxun-2 inhibited the growth of FOC TR4; (**B**) Pot control effect of strain Gxun-2 on FOC TR4. (**C**) Hyphae morphology of FOC TR4 on PDA plate and dual-culture plate under the scanning electron microscope. (**D**) Browning of banana seedling bulb profile. CG: control group; TG: treated group with Gxun-2.

After incubation at 28 °C for 7 days, Gxun-2 significantly inhibited the growth of FOC TR4 with a significant inhibition zone (Figure 1A,C), and the inhibition rate sometimes reached 75.25%. In addition, the observation results from scanning electron microscopy (SEM) showed the abnormal growth of hypha. The hypha of FOC TR4 without inoculation of Gxun-2 (control group, CG) grew vigorously with a uniform thickness and smooth texture. However, the hypha of FOC TR4 dual-culture with Gxun-2 (treated group, TG) grew disorderedly, with rupture, curves, knots, and swellings. Results show that Gxun-2 strain could inhibit the growth of FOC TR4 and change its hypha morphology.

The pot experiment showed that the incidence of banana *Fusarium* wilt when treated with Gxun-2 was significantly reduced, and this control effect can reach 84.21% (Figure 1B,D). Furthermore, the longitudinal section of banana bulbs in the control group (CG) was browned, but little browning was observed in the bulbs of the group treated with Gxun-2 (TG), indicating that Gxun-2 has an inhibitory effect on FOC TR4.

**Figure 2:**
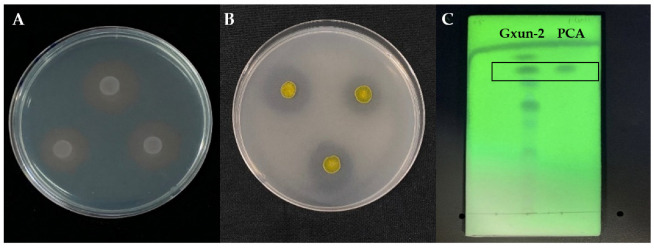
Plant growth-promoting and disease-prevention traits of the strain Gxun-2. (**A**) CAS test plate; (**B**) phosphate-solubilizing plate; (**C**) thin-layer chromatography. PCA: phenazine-1-carboxylic acid.

The strain Gxun-2 showed an evident orange halo on the CAS test plate (Figure 2A) and produced phenolate-type siderophores as well as phosphorus solubilizing activity (Figure 2B). The antifungal crude extract produced by Gxun-2 was collected after extraction and rotary evaporation. Thin-layer chromatography results showed four major spots under a 254 nm UV lamp, with one spot corresponding to the spot of the phenazine-1-carboxylic acid standard, with an Rf (retention factor) value of 0.83. Therefore, we deduced that the effective component of the substance with antagonistic activity produced by Gxun-2 was phenazine-1-carboxylic acid (Figure 2C).

### 2.2. Response Pattern of FOC TR4 to P. aeruginosa Gxun-2 Stress

In order to reveal the changes in the gene expression level of FOC TR4 after exposure to Gxun-2 stress, total RNA was extracted from an FOC TR4 dual-culture with Gxun-2 and wild-type FOC TR4 mycelia, respectively. Three replicates of the total RNA for the control group (CG) and the group treated with Gxun-2 (TG) were collected for the reverse transcription of mRNA into cDNA. More than 50 million clean reads were obtained after filtration. The error rate of each sample was less than 0.0271%, and the GC content was 51.61–53.48%. The Q20 and Q30 were greater than 97.2 and 93.26, respectively (Supplementary Table S1). The total map rate was higher than 80.03% in each sample (Supplementary Table S2), and the correlation coefficients were higher than 0.6048–0.8387 between TG and CG (Supplementary Table S3).

**Figure 3:**
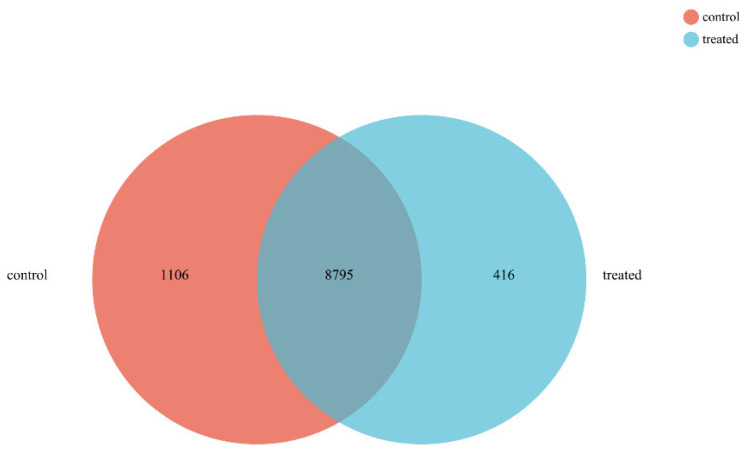
Venn diagram of gene differential expression. The sum of the numbers in each circle represents the total number of genes detected as expressed for that sample, and the overlapping portion of the circle indicates genes expressed in both samples tested.

**Figure 4:**
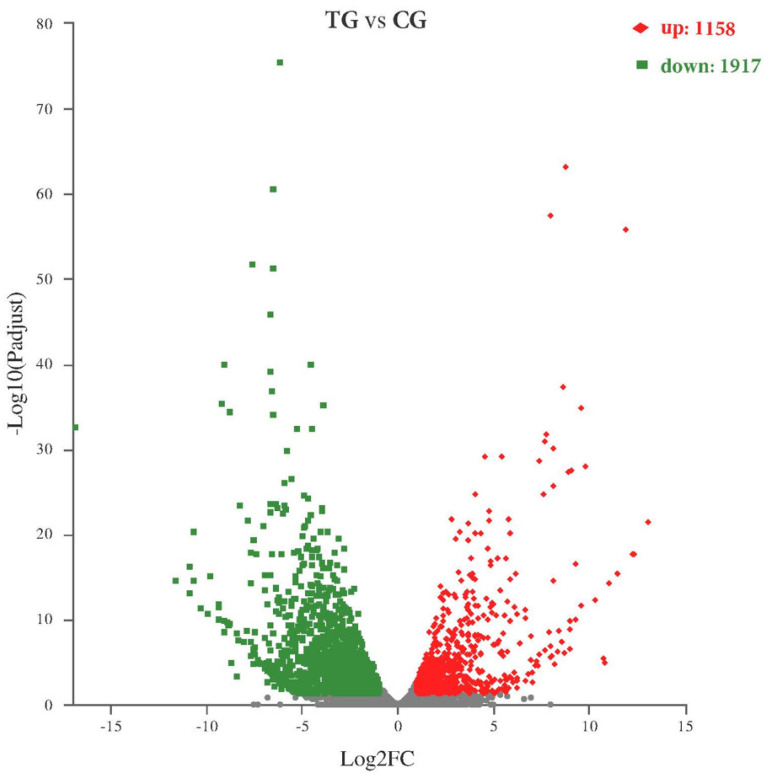
Volcano plot of DEGs between the treated group (TG) and the control group (CG). The y-axis corresponds to the mean expression value of log10 (*p*-value), and the x-axis displays the log2 fold change value. Grey dots: non-differential expressed genes. Red dots: up-regulated genes. Green dots: downregulated genes.

A total of 9901 expressed genes were collected in CG, while 9211 were collected in TG. The co-expressed genes between the two samples were 8795. Among them, 1106 and 416 genes were specifically expressed in control and treated, respectively (Figure 3). DESeq2 was used to test differentially expressed genes (DEGs) between the samples compared with CG, and 3075 genes in TG were significantly differentially expressed, including 1158 up-regulated and 1917 down-regulated genes (Figure 4).

### 2.3. Differential Expression Analysis and GO, KEGG Enrichment Analysis

**Figure 5:**
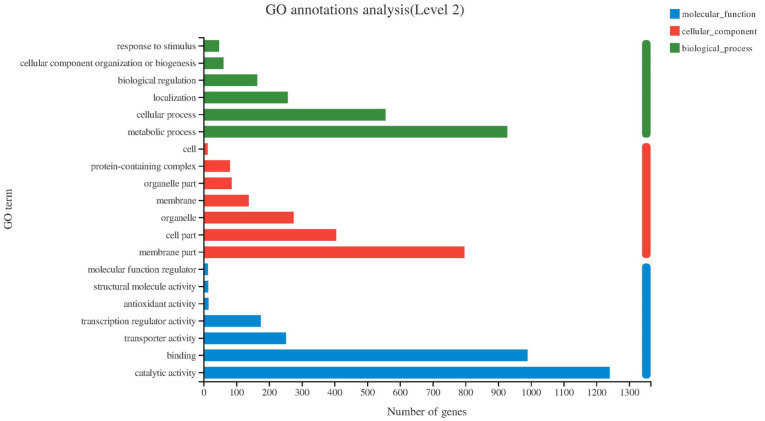
Gene ontology (GO) function classification of DEG.

Gene ontology (GO) was used to classify the functions of DEGs, including three basic categories, namely, biological process (BP), cellular component (CC), and molecular function (MF). On this basis, it can be divided into 30 subcategories, among which the three most populated terms in the BP category were biological (1292 genes), metabolic (927 genes) and oxidation–reduction process (272 genes). The integral component of membrane and the intrinsic component of membrane (790 genes) were the genes enriched in the CC category. The MF category mainly involved catalytic activity (1241 genes) and transferase activity (345 genes) (Figure 5).

**Figure 6:**
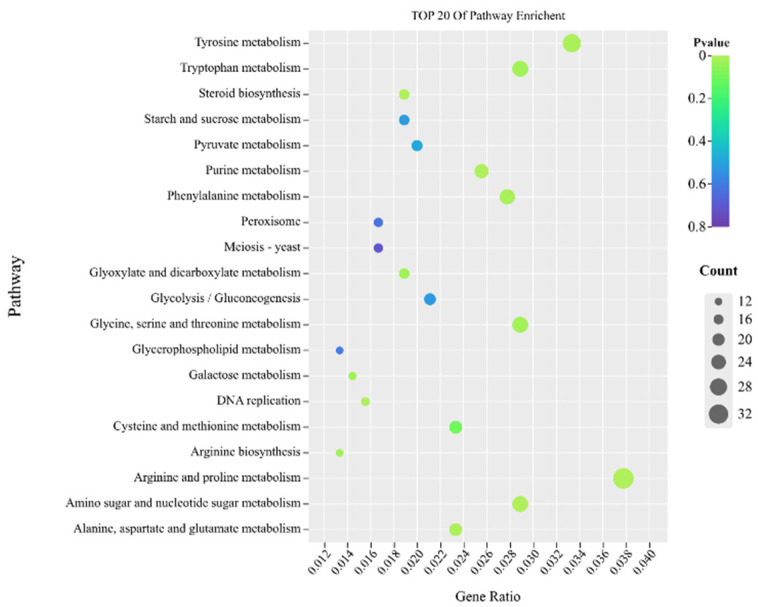
Bubble plot of the enriched KEGG pathways for DEGs. “Count” indicates the number of DEGs enriched in the pathway. “GeneRatio” indicates the ratio of enriched DEGs to background genes. The x-axis presents the percentage of DEGs belonging to the corresponding pathway. The y-axis represents the top 20 pathways.

The genomes of 3075 DEGs were further analysed using the Kyoto Encyclopedia of Genes and Genomes (KEGG) database, and 116 pathways were enriched in five branches, including metabolism, genetic information processing, environmental information processing, organic system, and cellular process. Among them, the DEGs enriched in the metabolism were dominant (213 genes). The main pathways were related with arginine and proline metabolism (map00330), tyrosine metabolism (map00350), amino sugar and nucleotide metabolism (map00520), glycine, serine and threonine metabolism (map00260), tryptophan metabolism (map00380), glycerophospholipid metabolism (map00564), steroid biosynthesis (map00100), and peroxidase bodies (map04146) enriched in KEGG (Figure 6).

### 2.4. Comparative Analysis of DEGs

#### 2.4.1. Antioxidant Activity-Related DEGs

Phenazine and its derivatives are the major determinants of biological control produced by *P. aeruginosa* (Figure 2B), and they can effectively inhibit fungal growth [12,13,14]. Phenazine can insert into a membrane and act as a reducing agent to transfer electrons to target cells, causing oxidative damage or even death by increasing superoxide free radicals in cells [15]. The antioxidant enzymes in FOC TR4 are the key enzymes that resist Gxun-2 stress. In the present study, two upregulated genes, namely, FOXG_15294 and FOXG_03076, were collected from the antioxidant system of the peroxisome pathway. These two upregulated genes may be related to the improved tolerance of FOC TR4 against oxidative damage and the repair of damage caused by oxidative free radicals [16,17].

ABC transporters are also a key detoxification factor, as they excrete toxic substances [18]. Five ABC transporter-related genes, namely FOXG_17197, FOXG_04837, FOXG_07972, FOXG_12952, and FOXG_15452, were significantly upregulated, which may help FOC TR4 to cope with the oxidative damage caused by phenazine.

#### 2.4.2. Cell Wall Synthesis-Related DEGs

Chitin, glucans, mannans, and glycoproteins are important components of the fungal cell wall [19]. In TG, the genes FOXG_10061, FOXG_05078, and FOXG_05290, which encode chitin synthetase, a class of glycosyltransferases that catalyse the synthesis of chitin, were downregulated in the amino sugar and nucleoside sugar metabolism pathways. Chitinase is mainly responsible for the catalytic decomposition of chitin, and five genes that encode chitinase (FOXG_09583, FOXG_12882, FOXG_00277, FOXG_11492, and FOXG_15329) were also downregulated in TG. The downregulated expression of these genes may allow for the fine-tuning of FOC TR4 under Gxun-2 stress.

The glucan in the fungal cell wall is mostly (1,3)-beta-glucan, whose deficiency in the cell wall will affect the growth of cells and eventually lead to cell rupture [20]. The gene FOXG_03721, related to the synthesis of (1,3)-beta-glucan in starch and the sucrose metabolism pathway, was significantly upregulated, while FOXG_01250, which is responsible for encoding (1,3)-beta-glucanase, was downregulated. The completely opposite expression of these two genes might be related to the reduction of (1,3)-beta-glucan consumption. Both fine-tuning processes are responses meant to mitigate cell wall damage.

#### 2.4.3. Cell Membrane Synthesis-Related DEGs

Fatty acids play an important role in forming the fungal plasma membrane and maintaining cell mobility [21]. In the present study, the DEGs (FOXG_16631, FOXG_05107, FOXG_16631, FOXG_05107, FOXG_05756, and FOXG_10933) related to fatty acid synthesis were all downregulated in TG. In addition, the gene FOXG_01555, which encodes fatty acid elongase, was upregulated, and this enzyme was mainly responsible for fatty acid chain extension. Sphingolipid is also an important component of fungal cell membranes. In the TG, six downregulated genes, namely, FOXG_00989, FOXG_05578, FOXG_15265, FOXG_10269, FOXG_02604, and FOXG_09964, were enriched in the sphingolipid metabolism pathway. The down-regulation of these genes will affect the function of the cell membrane, such as the transport of plasma membrane [22].

The sterols enriched in the plasma membrane are a critical lipid, which is an indispensable component of all eukaryotic cells [23]. Therefore, sterols can affect various membrane-related functions as a membrane component, such as maintaining the permeability and flow of the cell membrane [24]. In TG, 17 DEGs were identified in the steroid biosynthesis pathway, of which 13 genes (FOXG_11545, FOXG_02348, FOXG_08223, FOXG_03780, FOXG_06186, FOXG_03780, FOXG_06186, FOXG_15629, FOXG_01590, FOXG_10530, FOXG_09168, FOXG_04166, and FOXG_05355) were significantly upregulated in the ergosterol pathway. FOC TR4 increases the efficiency of ergosterol synthesis by up-regulating the genes mentioned above.

#### 2.4.4. Autophagy-Related DEGs

The autophagy pathway in fungi is involved in nutrient recycling under stress [25]. In the present study, five upregulated genes were related to autophagy in FOC TR4, namely FOXG_00582, FOXG_08160, FOXG_15833, FOXG_01950, and FOXG_10507, which encode autophagy-related proteins, namely Atg9, Sch9, Vps33, Vps45, and Pep4, respectively. These upregulated genes could improve the clearance of damaged cell structures and organelles in order to guarantee the normal growth of FOC TR4.

### 2.5. Validation of RNA-Seq Sequencing

**Figure 7:**
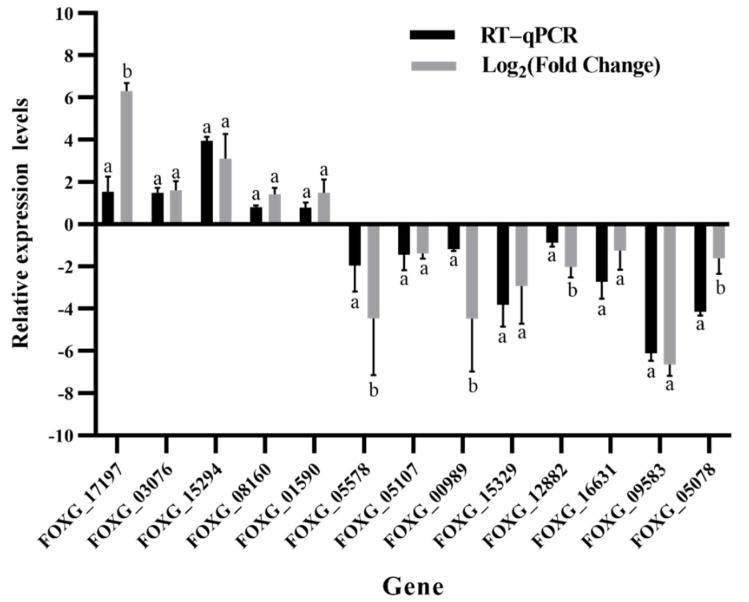
Relative expression level of 13 DEGs using the reference gene actin for normalization. Different lowercase letters in the table show significant differences via Duncan’s new complex polarization test (*p < 0.05*).

Thirteen DEGs for qRT-PCR were involved in the cell wall and membrane structure and antioxidant and autophagy of FOC TR4. These results are in agreement with the RNA-Seq high-throughput sequencing data (Figure 7), indicating a similar expression pattern of up- and downregulated genes to those in RNA-Seq sequencing, and they could be used for further analysis.

## 3. Discussion

**Figure 8:**
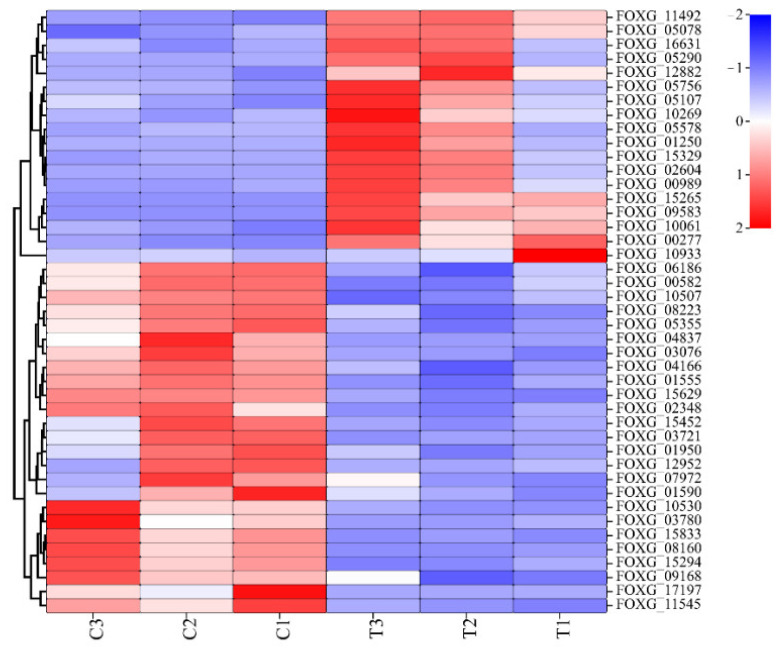
Heatmap of cell wall and membrane, antioxidant damage, and autophagy-related DEGs. The color scale indicates the counts of gene expression normalized by Z-score. “C1, C2, and C3” indicates the three replicates of the control group. “T1, T2, and T3” indicates the three replicates of the treatment group.

*P. aeruginosa* Gxun-2 could secrete various secondary metabolites, such as siderophores and PCA, which are essential to sustaining its ecological adaptability and survival. They could also inhibit the growth of a fungal pathogen through different antifungal mechanisms [26,27]. In addition, Gupta et al., demonstrated that *P. aeruginosa* could produce chitinase [28], while Peng and Sharon found that the metabolites of *P. aeruginosa* contained lipase and could inhibit the synthesis of fungal cell membrane and cell wall [29,30]. Therefore, the results of DEGs mainly focus on the significant changes in the synthesis of the cell wall and membrane, antioxidant damage, and autophagy in FOC TR4 cells (Figure 8).

In the present study, the growth of FOC TR4 was significantly inhibited under Gxun-2 stress. At the subcellular level, the growth of FOC TR4 hyphae dual-cultured with Gxun-2 was abnormal. Distortion, bifurcation, and knotting were observed, and they were possibly caused by antibacterial substances produced by Gxun-2. This result supports the hypothesis that hyphal growth is inhibited when fungi suffer from oxidative damage [31,32,33]. Peroxisomes are core organelles in eukaryotes where superoxide dismutase (SOD) and catalase (CAT) are both generated and detoxified [34]. Two genes, namely FOXG_15294 and FOXG_03076, of the antioxidant system in the peroxidase pathway, which encode CAT and superoxide dismutase 1 (SOD1), respectively, were upregulated. These two enzymes are important antioxidant enzymes in organisms that can repair the damage caused by free radicals [16,17]. When FOC TR4 is induced to produce oxidative stress by phenazine substances, superoxide free radicals in the cells increase, and the up-regulation of these two genes may be one of the important response reactions for FOC TR4 to reduce the damage under oxidative stress. Moreover, FOXG_04389, which encodes superoxide dismutase 2 (SOD2), was downregulated, possibly because SOD2 is a superoxide dismutase of Fe-Mn family [35], which needs to be combined with iron when catalysing the reaction. If the iron concentration in the cells’ changes, then the activity of SOD2 will be affected. The lack of iron in FOC TR4 can be attributed to the siderophore released by Gxun-2 and causes the decrease of SOD2 activity. Therefore, in response to oxidative damage, FOC TR4 downregulated the expression of SOD2 gene but upregulated the expression of the SOD1 gene of the Cu-Zn family. In addition, the ABC transporter is involved in the anti-oxidative stress response, and the ABC transporter has the detoxification function of expelling toxic substances [18]. The significant up-regulation of the five ABC transporter genes may coordinate the exclusion of oxidative radicals in response to the oxidative damage caused by phenazine substances.

The four genes encoding chitin synthase in the amino sugar and nucleoside sugar metabolic pathways were downregulated, and chitin synthase is a glycosyltransferase that catalyzes the synthesis of chitin [36]. The chitin-biosynthesis capability of FOC TR4 was decreased according to the downregulation of several genes. As a major component of the fungal cell wall, the insufficient synthesis of chitin may lead to changes in the structure and function of the cell wall. The genes FOXG_09583, FOXG_12882, FOXG_00277, FOXG_11492, and FOXG_15329, which encode chitinase are mainly responsible for decomposing chitin. These genes were downregulated, which possibly reduced the damage to the cell wall. The cell inner wall skeleton is composed of beta-glucan and chitin, playing the role of a flexible viscoelastic framework and determining the shape and strength of the cell wall to a large extent. The most abundant beta-glucan in the fungal cell wall is (1,3)-beta-glucan, which makes up between 65% and 90% of the whole beta-glucan content [37].

The antifungal metabolite produced by Gxun-2 could inhibit the synthesis of the cell wall and thus cause the cell wall to rupture. In this study, hypha curving and rupture were also observed. Therefore, FOC TR4 significantly upregulated the gene FOXG_03721 encoding (1,3)-beta-glucan synthase; a key enzyme in (1,3)-beta-glucan synthesis, FOC TR4 reduced cell wall damage by up-regulating (1,3)-beta-glucan synthase under the inhibition of Gxun-2. At the same time, FOXG_01250, which encodes (1,3)-beta-glucanase, was downregulated. (1,3)-Beta-glucanase mainly releases glucose by hydrolysing the non-reducing end of (1,3)-beta-glucan [38]. The upregulated gene FOXG_01250 in TG was possibly an attempt to alleviate the damage to the cell wall caused by the consumption of (1,3)-beta-glucan. Cell deformation and hyphae deformation occur when the chitin or dextran is inadequate to support the pressure of the contents [39]. The two processes above have completely opposite responses to Gxun-2 stress, suggesting that FOC TR4 alleviated the biological stressors by adjusting gene expression. However, the differential expression of these genes did not change the inhibitory state in FOC TR4 caused by Gxun-2. We still observed the abnormal growth of hyphae, such as bending and knotting, which supports that *P. aeruginosa* inhibits *Aspergillus fumigatus* growth by blocking (1,3)-beta-glucanase activity, thus altering the cell wall architecture [40].

Fatty acids are among the important fungal cell membrane structural components that maintain the morphological structure and biological function of the cell membrane. In the present study, one of the four DEGs encoding ELO3 was downregulated, a fatty acid chain elongase that is mainly responsible for fatty acid chain elongation [41]. The structure and function of the cell membrane of FOC TR4 may also be inhibited to some extent.

**Figure 9:**
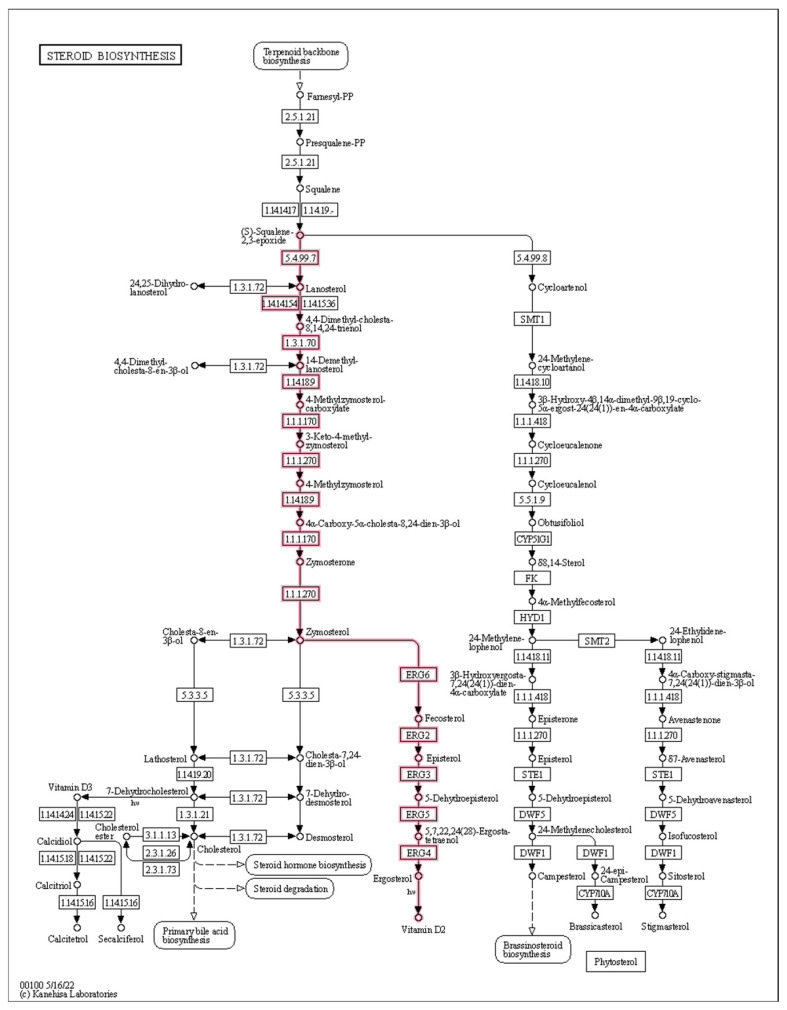
Analysis of DEGs related to steroid biosynthesis. The red highlights indicate higher expression (log_2_FC > 1) in treated group (TG).

Ergosterol (ERG), as a crucial part of the fungal cell membrane, is a critical sterol in the cell membranes of fungi. It is mainly responsible for maintaining the stability and fluidity of the cell membrane [39,42]. ERG biosynthesis is tightly regulated by 25 known enzymes along the ERG production pathway. Among them, Erg11 and Erg5 are two crucial enzymes in ergosterol synthesis. The expression levels of two key genes (FOXG_13138 and FOXG_04166) that encode Erg11 and Erg5 were upregulated through ergosterol synthesis in FOC TR4. The activities of these two enzymes decreased in FOC TR4 under the stress of iron deficiency. Thus, FOC TR4 up-regulates these genes in order to mitigate the damage from iron deficiency. Two fungicides, namely terbinafine and naftifine, target ergosterol synthase and eventually lead to the cracking and death of fungal cells [43]. The ergosterol synthesis pathway depends on iron in four enzymatic steps, which include the two enzymes Erg5 and Erg11 encoded by FOXG_04166 and FOXG_11545. Erg5 catalyses the biosynthesis of ergosta 5, 7, 22, 24 (28)-trienol, while 4,4-dimethyl-ergosta 8, 14, 24 (28)-trienolis, the direct product catalysed by Erg11. These two iron-dependent reactions are mainly involved in the process of ergosterol synthesis, and are responsible for the second and penultimate reactions of the ergosterol biosynthesis pathway (Figure 9). Siderophores produced by Gxun-2 are natural iron chelators with low molecular secondary metabolites between 200–2000 Da and a high affinity for Fe^3+^ [44]. They are secreted in order to acquire iron as needed by *P. aeruginosa*, which results in reduced available iron in the environment. The activities of these two iron-dependent enzymes in FOC TR4 are also reduced, leading to the accumulation and depletion of the compounds of the intermediate steps, respectively. Shakoury-Elizeh et al., found that compared with iron-replete cells, iron-deficient cells exhibited a 24- and 3.3-fold accumulation of squalene and lanosterol, respectively. They also exhibited depletion of the major oxysterols, namely zymosterol (3-fold) and ergosterol (2-fold) [45].

Autophagy is a ubiquitous and non-selective degradation process in eukaryotic cells which is conserved from yeast to human. It is a physiological mechanism that promotes the turnover of cell macromolecules and organelles through a lysosomal degradative pathway in order to maintain cell homeostasis. Five upregulated genes are related to the autophagy pathway in TG. Among them, ATG 9 is an interleaflet lipid transport protein that drives autophagy [46]. Sch 9 signaling pathways regulate the induction of autophagy in yeast [47]. Vps33 is involved in multiple autophagy-related pathways of vesicular trafficking to the vacuole and in the maintenance of vacuolar integrity [48]. Vps45 is required for membrane fusion in autophagy [49]. Pep4 is a proteinase A that limits the proteolytic capacity of the vacuole in a substrate-dependent manner [50]. The upregulated genes above may increase the phagocytosis and degradation of the oxidative material in order to reduce oxidative damage as a response of FOC TR4 when damaged by the antifungal substances secreted by Gxun-2.

**Figure 10:**
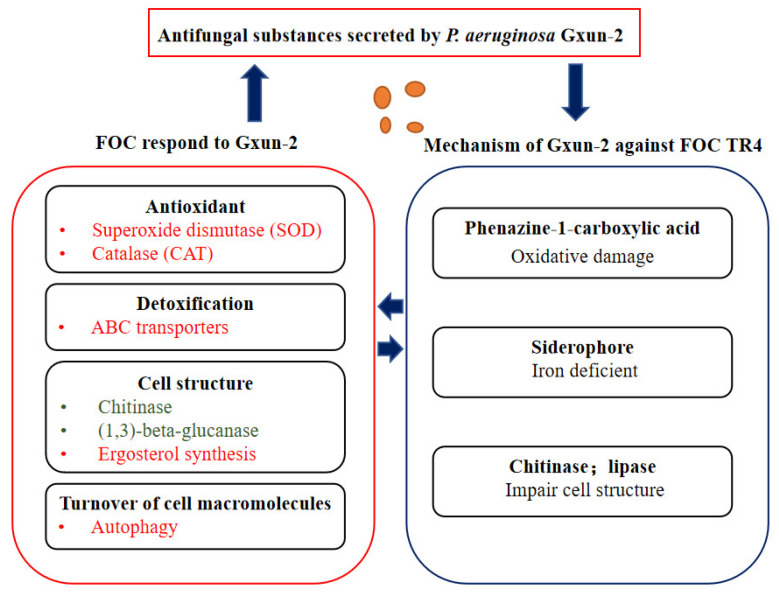
Pattern of interaction between FOC TR4 and Gxun-2.

According to the comparative transcriptome analysis, FOC TR4 regulated multiple genes in various pathways in order to reduce the damage to the fungal cell wall, cell membrane, and the oxidative compounds toxic to cells caused by antifungal substances, including siderophore, phenazine, and chitinase produced by *P. aeruginosa* Gxun-2 (Figure 10).

## 4. Materials and Methods

### 4.1. Strains and Culture Conditions

*P. aeruginosa* Gxun-2 was isolated from the rhizosphere of healthy banana in Guangxi province, China. It is preserved in the Guangdong Microbiol Culture Collection Center (GDMCC No.61615). *Fusarium oxysporum* f.sp. *cubense* with Tropical Race 4 (FOC TR4 TR4) and other pathogenic fungi were obtained from the Guangxi Academy of Agricultural Sciences, Nanning, China. They were cultured on potato dextrose agar (PDA) medium at 30 °C for 7 d.

### 4.2. Inhibition of P. aeruginosa Gxun-2 to F. oxysporum and Its Mechanism

The inhibitory effect of Gxun-2 on the hyphal growth of FOC TR4 was tested using the co-culture method. First, FOC TR4 was inoculated into the center of PDA plate, and streaks of Gxun-2 four 7.0 cm in length were inoculated at a distance of 2.5 cm from the FOC TR4. The PDA plate inoculated with FOC TR4 was used as the control. Three replicates were set for each group, and the cells were incubated at a constant temperature of 28 °C for 7 days [10]. The inhibitory effect of the antagonistic strain on the pathogenic fungus was observed in order to calculate the inhibition rate, and the morphology of the hyphae at the colony edge of the pathogenic bacteria was observed under a scanning electron microscope (SEM).

Inhibition of mycelial radial rate = Σ (average diameter of target colony in control group-average diameter of target colony in experimental group)/average diameter of target colony in control group × 100% [51].

The pot experiment was conducted following the methods of Chen et al.; the disease grade was determined according to the browning degree of the longitudinal section of the banana seedling corm. Grade 0: no browning occurred in the longitudinal section of the corm; Level 1: browning area in longitudinal section of corm ≤ 25%; Level 3: 25% < browning area in longitudinal section of bulb ≤ 50%; Level 5: 50% < browning area in longitudinal section of bulb ≤ 75%; Level 7: browning area of corm longitudinal section > 75% [52]. Disease index = Σ (condition level × number of diseased plant of this level)/(the highest level of disease × total number of disease); Control effect (%) = (CG disease index − treatment disease index)/CG disease index × 100% [53].

The siderophores were detected using the chrome azurol S (CAS) solid medium [54], and Arnow and ferric perchlorate assays were used to detect catechol siderophores and hydroxamates [55,56]. Phenazine -1- carboxylic acid (PCA) was isolated and extracted as described by Palchevskaya et al. [57]. Analysis of components was conducted on the basis of Rf (retention factor) values [58].

### 4.3. Preparation of FOC TR4 Mycelium and RNA Extraction

The confrontation experiment of Gxun-2 and FOC TR4 was carried out as described above, and the medium was incubated at a constant temperature of 28 °C for 7 days. The mycelia from the Gxun-2-exposed boundary of the dual-culture PDA plate were collected and labelled as the treatment group (TG), while the mycelia collected from wild-type FOC TR4 were labelled as control group (CG). Both the TG and CG had three biological replicates. Collected samples were lyophilised with liquid nitrogen and stored at −80 °C for RNA extraction.

### 4.4. Preparation of FOC TR4 Mycelium and RNA Extraction

Total RNA was extracted from the tissue using TRIzol^®^ Reagent according to the manufacturer’s instructions (Invitrogen, Waltham, MA, USA), and genomic DNA was removed using DNase I (TaKara, Shanghai, China). Then, RNA quality was determined using 2100 Bioanalyser (Agilent Technologies, Santa Clara, CA, USA) and quantified using ND-2000 (NanoDrop Technologies, Waltham, MA, USA). An RNA-seq transcriptome library was prepared following the instructions of the TruSeqTM RNA sample preparation kit from Illumina (San Diego, CA, USA). The processing of original images to sequences, base-calling, and quality value calculations were performed using the Illumina GA Pipeline (version 1.6) by Shanghai Majorbio Bio-pharm Technology Co., Ltd. (Shanghai, China), in which 150 bp paired-end reads were obtained.

### 4.5. Differential Expression Analysis, GO, and KEGG Enrichment Analysis

GO pathway enrichment analysis was performed using Goatools software, (https://github.com/tanghaibao/Goatools) (accessed on 8 July 2022) and the KEGG pathway enrichment analysis was performed using KOBAS (http://kobas.cbi.pku.edu.cn/home.do) (accessed on 8 July 2022) via Fisher’s exact test. *p* -values were corrected using four multiple tests (Bonferroni, Holm, Benjamini and Hochberg FDR, and Benjamini Yekutieli), and the genes were considered to be differentially expressed when FDR ≤ 0.05.

### 4.6. RT-qPCR Assay

In order to verify the reliability of DEGs from the transcriptome sequencing of FOC TR4, we selected 13 genes for qRT-PCR validation with the actin gene as the internal reference gene. Primer (Version 5.0, Davis, CA, USA) was used to design the primers of 13 selected DEGs. Primer (Version 5.0) was used to design the primers of 13 selected DEGs (Table 1). PCR amplification was performed using the BIO-RAD system (Hercules, CA, USA), and the expression analysis was carried out using built-in software. The reaction system consisted of cDNA 1 μL, 10 μM forward PCR primer 0.5 μL, 10 μM reverse PCR primer 0.5 μL, BlasTaqTM 2X qPCR MM1 10 μL, and nuclease-free H_2_O 8 μL, totaling 20 μL. The PCR program was as follows: 95 °C for 180 s, 40 cycles of (95 °C for 15 s, 60 °C for 1 min). A dissolution curve was then generated. The qPCR for each gene was repeated three times, and the average (Ct) was calculated. The relative expression level of each gene was calculated using the 2-ΔΔCt method [59].

## 5. Conclusions

In summary, the soil bacterium *P. aeruginosa* Gxun-2 was a strong inhibitor of FOC TR4 growth both in vitro and in vivo, resulting in the significant control of banana *Fusarium* wilt. An examination of FOC TR4 exposed to Gxun-2 showed damage to cell walls and membranes. Comparative transcriptome analysis showed that FOC TR4 can respond and attempt to alleviate damage caused by *P. aeruginosa*. It attempted to increase cell wall and membrane synthesis, antioxidant responses, detoxification, and export of antifungal substances from its cells. However, these processes were unable to prevent cell damage, and their ability to cause *Fusarium* wilt was greatly compromised with Gxun-2. These results lay the foundation for comprehensively expounding the mechanism of interaction between FOC TR4 and *P. aeruginosa*. They also provide an effective strategy for the control of *Fusarium* wilt in banana.

## Figures and Tables

**Table 1:** Primer sequences of differentially expressed genes in *Fusarium oxysporum* for qRT-PCR analysis.

Gene ID	Function	Primer Sequence (5′ to 3′)	Product Size (bp)
FOXG_01590	C-8 sterol isomerase	F: GCTCCTCCTGGTTCTCGGTCTC R: TCCTCTCCGTGCTTAGCGATGG	144
FOXG_05578	Arylsulfatase	F: CCGATGACCTTGGCTTCAGTGATG R: GCCTGAGCGGTATGGAAGTTGG	115
FOXG_05107	Conserved hypothetical protein	F: CTGACATCAAGCCTGAGGACTTCAC R: GGAGCGGTCTGTGGGTAGGTAG	104
FOXG_08160	Hypothetical protein similar to mitochondrial RNA splicing protein	F: CACAGCTCTACAGTTCCTCGCATAC R: GACCAGCAGCAAAGCCACCAG	119
FOXG_00989	Arylsulfatase	F: CGATGATGGTCCAGCAGCCTTG R: TTCCTCGCCGACTCAGCCTTC	147
FOXG_15329	Conserved hypothetical protein	F: CGGGCGTCATGGTGCTAAGTG R: CGTATCAGCGTCGTCATAGCCTATC	137
FOXG_12882	Endochitinase 1 precursor	F: GCCTACGATTATGCTGGGTCTTGG R: TGGCGGCGGATGTAGAGAAGG	106
FOXG_16631	Conserved hypothetical protein	F: TGGACCAGTGCAGAGCGATTTATTG R: CTGAAGCCAACCAGCAACAATCTG	134
FOXG_09583	Chitinase 1 precursor	F: CTGAGGAGGCGACGGGTAGTG R: GTCAGTTGCGATGCGGGAAGG	100
FOXG_05078	Chitin synthase class I	F: ATACCAAGACCAACCGCAACAAGG R: GCAGAACTGGAGGAACAGGACAATC	113
FOXG_17197	Conserved hypothetical protein	F: GTTGGACATACCTGGCGTGGATTC R: TCCTCCGTGGCTGTTCCGTAG	102
FOXG_03076	Superoxide dismutase	F: CTTCCGCTGGTCCTCACTTCAAC R: GGTGACAGAGCCCTTAGCATTTCC	128
FOXG_15294	conserved hypothetical protein	F: AGGGATCTGTGGAGGAGAGTCTTTG R: AGGAGCAGGTGTACCAGCCAAG	90

## Data Availability

The data presented in this study are available on request from the corresponding author.

## Supplementary Materials

The following supporting information can be downloaded at: https://www.mdpi.com/article/10.3390/ijms232315432/s1, Table S1: Sequencing data Statistics of *F. oxysporum* with and without *P. aeruginosa*; Table S2: Spearman’s correlation coefficients of *F. oxysporum* with and without *P. aeruginosa* Gxun-2 suppression; Table S3: Correlation coefficients of different samples.
