# Supplementary material for: Comparative Transcriptome Analysis Unravels the Response Mechanisms of Fusarium oxysporum f.sp. cubense to a Biocontrol Agent, Pseudomonas aeruginosa Gxun-2
Source: Int J Mol Sci. 2022 Dec 6;23(23):15432. doi: 10.3390/ijms232315432 (PMC9735772; doi:10.3390/ijms232315432)
Supplement: Supplementary file 1 [file ijms-23-15432-s001.zip › ijms-2064202-supplementary.pdf]

Supplementary Table S1 Sequencing data Statistics of *F. oxysporum* with and without *P. aeruginosa* Gxun-2 suppression

Sample	Raw reads	Raw bases	Clean reads	Clean bases	Error rate(%)	Q20(%)	Q30(%)	GC (%)
CK1	55389296	8363783696	53344044	7752307162	0.0236	98.57	95.72	53.2
CK2	56741986	8568039886	54874222	7975658072	0.0233	98.65	95.97	53.42
CK3	53597812	8093269612	51635362	7477765082	0.0235	98.6	95.79	53.48
TR1	89384210	13497015710	54564184	5696082232	0.0271	97.2	93.26	51.61
TR2	57366034	8662271134	55362162	7982154280	0.0236	98.55	95.68	53.26
TR3	56240414	8492302514	54308844	7874779049	0.0234	98.64	95.97	53.48

Supplementary Table S2 Spearman's correlation coefficients of *F. oxysporum* with and without *P. aeruginosa* Gxun-2 suppression

Sample	Total reads	Total mapped	Multiple mapped	Uniquely mapped
CK1	53344044	45840005(85.93%)	626454(1.17%)	45213551(84.76%)
CK2	54874222	47683029(86.9%)	823648(1.5%)	46859381(85.39%)
CK3	51635362	45155927(87.45%)	767117(1.49%)	44388810(85.97%)
TR1	54564184	43666607(80.03%)	1078099(1.98%)	42588508(78.05%)
TR2	55362162	47624872(86.02%)	738776(1.33%)	46886096(84.69%)
TR3	54308844	47365981(87.22%)	830669(1.53%)	46535312(85.69%)

Supplementary Table S3 correlation coefficients of different samples

	CK1	CK1	CK1	CK1	CK1	CK1
CK1	1	0.8622	0.8124	0.6212	0.697	0.7028
CK2	0.8622	1	0.9395	0.7137	0.6108	0.6048
CK3	0.8124	0.9395	1	0.8387	0.7136	0.685
TR1	0.6212	0.7137	0.8387	1	0.8193	0.7468
TR2	0.697	0.6108	0.7136	0.8193	1	0.9396
TR3	0.7028	0.6048	0.685	0.7468	0.9396	1
